# Effect of bioactive glass addition on the physical properties of mineral trioxide aggregate

**DOI:** 10.1186/s40824-021-00238-2

**Published:** 2021-11-24

**Authors:** Jei Kim, Hyun-Jung Kim, Seok Woo Chang, Soram Oh, Sun-Young Kim, Kyoung-Kyu Choi, Duck-Su Kim, Ji-Hyun Jang

**Affiliations:** 1 Department of Conservative Dentistry, Graduate School, Kyung Hee University, Seoul, South Korea; 2 Department of Conservative Dentistry, Kyung Hee University Dental Hospital, Seoul, South Korea; 3 Department of Conservative Dentistry, School of Dentistry, Kyung Hee University, 26 Kyungheedae-ro, Dongdaemun-gu, Seoul, 02447 Republic of Korea; 4 Department of Conservative Dentistry and Dental Research Institute, School of Dentistry, Seoul National University, Seoul, South Korea

**Keywords:** Mineral trioxide aggregate, Bioactive glass, Pushout bond strength, Compressive strength, Set-ting time, X-ray diffraction analysis

## Abstract

**Background:**

The addition of bioactive glass (BG), a highly bioactive material with remineralization potential, might improve the drawback of weakening property of mineral trioxide aggregates (MTA) when it encounters with body fluid. This study aims to evaluate the effect of BG addition on physical properties of MTA.

**Methods:**

ProRoot (MTA), and MTA with various concentrations of BG (1, 2, 5 and 10% BG/MTA) were prepared. Simulated body fluid (SBF) was used to investigate the effect of the storage solution on dentin remineralization. Prepared specimens were examined as following; the push-out bond strength to dentin, compressive strength, setting time solubility and X-ray diffraction (XRD) analysis.

**Results:**

The 2% BG/MTA showed higher push-out bond strengths than control group after 7 days of SBF storage. The 2% BG/MTA exhibited the highest compressive strength. Setting times were reduced in the 1 and 2% BG/MTA groups, and solubility of all experimental groups were clinically acceptable. In all groups, precipitates were observed in dentinal tubules via SEM. XRD showed the increased hydroxyapatite peaks in the 2, 5 and 10% BG/MTA groups.

**Conclusion:**

It was verified that the BG-added MTA increased dentin push-out bond strength and compressive strength under SBF storage. The addition of BG did not negatively affect the MTA maturation reaction; it increased the amount of hydroxyapatite during SBF maturation.

## Introduction

Mineral trioxide aggregate (MTA) was introduced in 1993 [1] and has been used in the repair of root perforations, capping of pulps with reversible pulpitis, apexification, and as a root-end filling material, because of its sealing ability and biocompatibility [2]. The main component of the MTA powder is calcium silicate, which is formed by the reaction of CaO and SiO_2_. Through the hydration process with distilled water (DW), MTA forms calcium silicate hydrate and calcium hydroxide in the form of a crystalline phase in the set MTA, followed by hydroxyapatite (Ca_10_ (PO_4_)_6_ (OH)_2_) formation, which has a sealing effect [1]. MTA has been accepted as a suitable endodontic restorative material as it provides favorable histologic and clinical outcomes, because of its effective sealing and biocompatibility [3, 4]. However, several studies have presented the shortcomings of MTA [2, 4–14], including long setting time [1, 2, 5–7], poor handling properties [6, 7] and poor physical properties when interacting with body fluids (BF) [8, 9]. Methods such as adding additives (calcium chloride [10], sodium hydrogen phosphate [12], methylcellulose [10], bioactive glass (BG) [11] or changing the hydration liquid to calcium chloride [7], calcium lactate gluconate [7], and elastin-like polypeptide [6] have been attempted to overcome the disadvantages of MTA; these methods have demonstrated improvements in some properties of MTA.

In most clinical application environments of MTA, more than one surface of the MTA material is in contact with the periodontal or pulpal tissue. When the unset MTA is contaminated with tissue fluid (e.g., blood and interstitial fluid from dental pulp and periodontal tissues), the mechanical properties of MTA are adversely affected [15]. Previous investigations reported that blood contamination has detrimental effects on the resistance of displacement [8], microhardness, compressive strength, and surface microstructure [9]; further, it is associated with changes in the setting reaction of MTA with lower formation of calcium hydroxide crystalline structures [16]. Thus, additives and alternative liquid solutions to improve the properties of MTA need to be addressed in clinically applicable conditions.

In 1969, Dr. Larry Hench introduced BG for the first time; it was an alternative to nearly inert implant materials [17]. It is a highly bioactive material with silicon dioxide as its main component. When it encounters water from BF or simulated body fluid (SBF), BG immediately undergoes ionic dissolution and glass degradation [17]. It releases silica ions to form a silica-rich layer on the surface. Outside this layer, calcium and phosphoric acid from the BF form a layer of calcium phosphate, which becomes hydroxyapatite when it crystallizes [18]. Further, BG can bond to mineralized and soft tissue [19]; this leads to a superior surface area with a higher dissolution rate and faster apatite formation. In addition, it improves the mechanical properties of such composites for natural bones [18].

Owing to the ability of BG to form apatite, several investigations have been conducted in the recent years to use BG as a remineralization additive in dental restoration materials owing to its ability to form apatite [11, 20]. Jang et al. [21] reported that dental composite resins including BG remineralize surrounding dentin; Kim et al. [22] suggested that a glass ionomer including BG had the same effect. Thus far, there have been a few studies on the addition of BG to MTA; among them, the physical properties of MTA supplemented with BG, which are required for the evaluation of endodontic restorative materials, have been rarely investigated. This study aims to evaluate the effect of BG addition on the physical properties of MTA, which includes push-out bond strength to dentin, compressive strength, setting time, solubility, and X-ray diffraction (XRD) analysis.

## Materials and methods

### Material preparation

For specimen preparation, DW was used as the liquid solution in all experimental groups. The group names were labeled according to the type of powder used to prepare the MTA specimens. White ProRoot MTA (Dentsply Sirona, Tulsa, USA) was used as the control and labeled as MTA. The major constituents of MTA powder were tricalcium silicate (Ca_3_SiO_5_), dicalcium silicate (Ca_2_ SiO_4_), tricalcium aluminate (Ca_3_Al_2_O_6_), bismuth oxide (Bi_2_O_3_) and calcium sulfate (CaSO_4_). The MTA with BG supplemented groups (BG/MTA groups) were prepared using the MTA and 63S BG (Bonding Chemical, USA) at four different concentrations: 1, 2, 5, and 10 wt%; these were labeled as 1% BG/MTA, 2% BG/MTA, 5% BG/MTA, and 10% BG/MTA, respectively. The composition of 63S BG was 63% SiO_2_, 31% CaO, and 6% P_2_O_5_, and its particle size was less than 20 μm. The L/P ratio of DW to the powder was 0.3 throughout the experiments, which was set according to the manufacturer’s recommendations.

### Preparation of storage solutions

27 mM HCO_3_- Tris SBF with pH 7.4 was used to investigate the effect of the storage solution on dentin remineralization. The SBF was prepared as described by Tas et al [23] The composition of Tris SBF is listed in Table 1.

**Table 1:** Compositions of 27 mM HCO_3_- Tris SBF^a^

Composition	Amount (g/L)
NaCl	6.547
NaHCO_3_	2.268
KCl	0.373
Na_2_HPO_4_∙2H_2_O	0.178
MgCl_2_∙6H_2_O	0.305
CaCl_2_∙2H_2_O	0.368
Na_2_SO_4_	0.071
(CH_2_OH)_3_CNH_2_	6.057

^a^*SBF* Simulated body fluid

### Push-out bond strength

Extracted caries-free human third molars were selected for this study. The included teeth were obtained from patients whose teeth were indicated for extraction, and informed consent was obtained. The experimental protocol using human teeth was reviewed and approved by the Kyung Hee University Institutional Review Board (KHU-1808-1), and all methods were performed in accordance with the Declaration of Helsinki guidelines and regulations. The midcoronal portion of the dentin of the tooth was horizontally cut into 2-mm-thick sections using a water-cooled high-speed diamond bur (*n* = 10). A cylindrical cavity (1.5 mm diameter) was prepared in each dentin disc with a depth-cutting bur (Microcopy, Kennesaw, GA). The dentin discs were soaked sequentially in 17% ethylene diamine tetraacetic acid (EDTA) and 2.5% sodium hypochlorite for 1 min each, washed with phosphate buffered saline, and dried. Each matrix was mixed with DW and carefully loaded into the cavity without compaction pressure. The specimens were stored at 37 °C under 95% humidity for 48 h [24] and stored in SBF for 3, 7, and 14 days in a 37 °C cabinet [13]. The SBF solution was replaced every 2 days to prevent autogenous precipitation.

After each storage period, the push-out bond strength was measured using a universal testing machine (AGS-X; Shimadzu, Tokyo, Japan) at a 1.0 mm/min (diameter 1 mm) crosshead speed. The maximum load applied to the specimens in the cavity before dislodgement was recorded in Newtons. The push-out bond strength values in megapascals were calculated by dividing the maximum load by the total contact area of the cavity in mm^2^ [25].

### FE-SEM analysis

The two remaining samples from each group after the push-out bond strength test (7 days of SBF storage) were placed individually in plastic containers containing 15 ml of SBF (pH = 7.4), and they were then stored for an additional 14 days at 37 °C to analyze the mixture-dentin interface [13]. The SBF solution was replaced every 2 days to prevent autogenous precipitation.

After 14 days of storage in SBF, the specimens were washed in DW and processed for FE-SEM observations. The specimens were cut along the center to expose the internal interface between the MTA and the dentin wall using a high-speed diamond bur under water irrigation. The exposed internal surfaces were serially polished with 600 grit SiC papers. The smear particles were removed by soaking in a 17% EDTA solution for 1 min. The specimens were dried at ambient temperature for 24 h. The interfaces were then observed under a FE-SEM (HITACHI S-4700, at an accelerating voltage of 10 kV, Tokyo, Japan) after Pt sputter coating.

### Compressive strength

The compressive strength was determined using the ISO 9917-1 method [26, 27]. Each material was mixed and placed in an acrylic mold (4.0 mm inner diameter and 6.0 mm height) [5]. After the placement, the complete assembly was transferred to a cabinet maintained at 37 °C under 95% humidity for a day for complete setting. The specimens were removed from the molds and checked visually for air voids or chipped edges. All defective specimens were discarded.

The specimens were stored in SBF solution for 7 days and maintained at 37 °C [28–30]. The SBF solution was replaced every 2 days to prevent autogenous precipitation.

The specimens were tested using the universal testing machine at a crosshead speed of 1 mm/min. The maximum load required to fracture each specimen was measured, and the compressive strength (C) was calculated in megapascals using

$$ \mathrm{C}=4\mathrm{P}/{\uppi \mathrm{D}}^2, $$

where P denotes the maximum load (N), and D denote the mean diameter of the specimen (mm).

### Etting time

The setting time was determined according to the International Organization for Standardization (ISO) 9917–1 method using a Gillomore apparatus (Heungjin Co. Ltd., Gyeonggi-do, Korea) [26, 27]. A total of three specimens were prepared in a circular acrylic mold (inner diameter = 10 mm; height = 5 mm). The assembly was placed in a cabinet at 37 °C and 95% humidity.

The final setting time was measured using a 1-lb heavy needle with a cylindrical tip (diameter = 1.06 mm) of the Gillmore apparatus. After mixing for 90 s, the indenter was carefully loaded vertically onto the surface of the specimens and allowed to remain there for 5 s. The indentations were repeated at 30 s intervals until the needle failed to make a visible indentation in the test specimen to determine the approximate setting time. This process was repeated starting the indentation at 30 s before the approximate setting time was determined, and indentations were made at 10 s intervals. This test was repeated five times for each specimen.

### Solubility

The solubility was assessed according to the ISO 6876 standard [27, 31]. A total of eight specimens were evaluated to examine their solubility. Each material was mixed and placed in split-ring molds (internal diameter = 20 ± 1 mm and 1.5 ± 0.1 mm). The filled molds were placed in a cabinet maintained at 37 °C and 95% humidity for a day. The specimens were removed from the molds and the weight before the DW storage of each specimen was determined to the nearest 0.001 g using a precision scale (AX200, SHIMADZU, Japan; precision = 0.0001 g).

The specimens were placed in a shallow dish, and 50 ± 1 mL of DW was added. The dish was covered and placed in a cabinet maintained at 37 °C for a day. Then, the specimens were removed and washed with 2–3 mL of fresh DW on a shallow dish. The water in the dish was evaporated without boiling and dried to a constant mass at 110 ± 2 °C. The dishes were weighed after cooling. The differences between this weight and the original dish weight were divided into the initial weight of the specimens and multiplied by 100. The results were recorded as the solubility of the specimen. This test was repeated four times for each group.

### XRD analysis

The XRD analysis was performed to compare the compositions of the reactants. Each material was mixed and placed in a rubber mold (inner diameter = 7.0 mm; height = 2.5 mm), and it was allowed to fully set during incubation at 37 °C in a 95% humidity cabinet for 24 h [29]. After the setting, the specimens were removed from the molds.

Immediately after setting, half of the spectra (*n* = 2) for each group were examined via XRD using an X-ray diffractometer (PANalytical B. V., Almelo, Netherlands) with CuKa radiation within a 2θ range of 5–40° at a scanning speed of 1.2° min − 1. The Cu X-ray source was set at an accelerating voltage of 45 kV and a current in the electron beam at 40 mA.

The remaining specimens were examined after 7 days of SBF storage. The specimens were individually stored in SBF using polyethylene containers for 7 days at 37 °C [30]. The structures of the set specimen and surface deposits formed in the SBF assays were examined using XRD analysis. The diffraction peak positions were determined using the peak-fitting program (Origin v7.5) software.

### Statistical analysis

The push-out bond strength and compressive strength of groups were analyzed statistically by two-way ANOVA to determine the interaction between variables; one-way ANOVA with Bonferroni test was performed for post hoc test for each variable. The data of the solubility were statistically analyzed using one-way ANOVA with Bonferroni post hoc comparison, respectively. Differences were considered statistically significant at *p* < 0.05. All statistical analyses were performed using SPSS (version 22.0; IBM Corp., Armonk, NY).

## Results

### Push-out bond strength

**Fig. 1:**
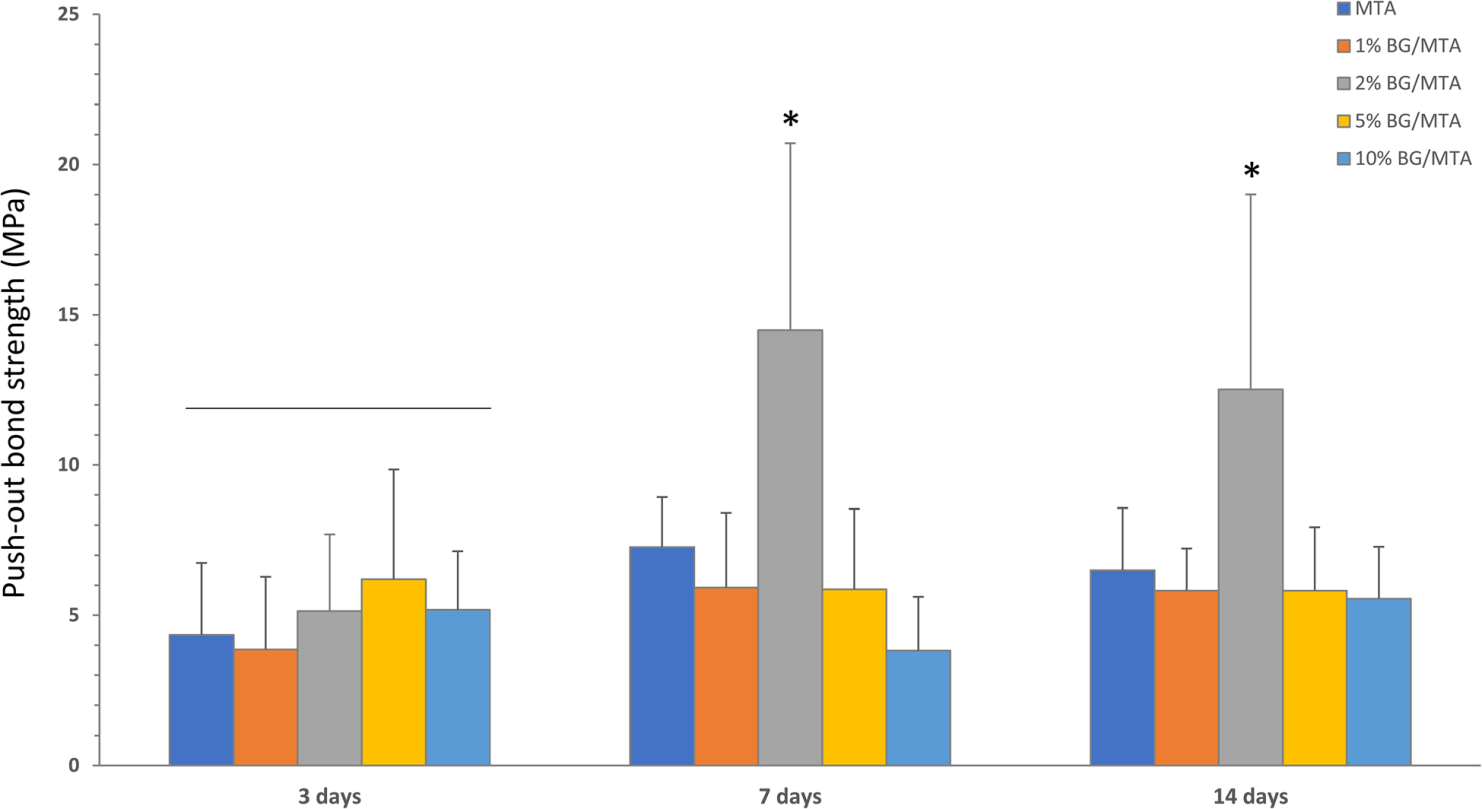
Results of the push-out bond strength. The results of the experimental groups were compared at 3, 7, and 14 days as storage periods. * indicates a statistically significant difference between materials (*p* < 0.05)

Figure 1 show the push-out bond strength results for all experimental groups. The 2% BG/MTA group showed a significantly higher value than the control group after 7 days of SBF storage (*p* < 0.05). In the 1 and 2% BG/MTA groups, the strength of the push-out bond increased as the storage period increased (*p* < 0.05) (Fig. 1), whereas the 5 and 10% BG/MTA groups did not show significant differences with time.

### FE-SEM analysis

**Fig. 2:**
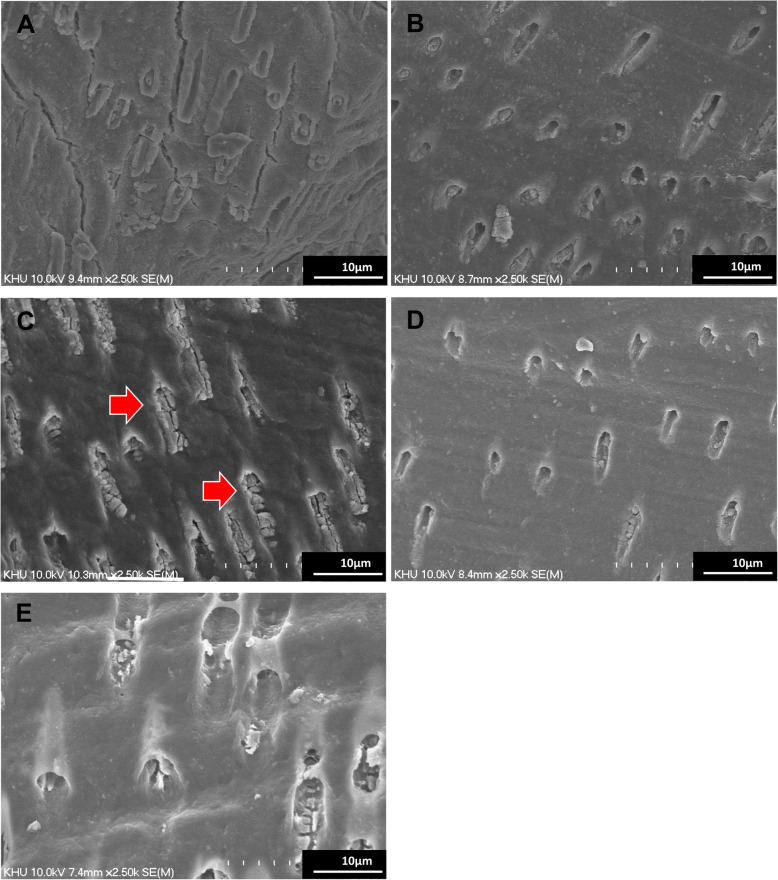
Representative FE-SEM images of the dentin-MTA interface: 2500 magnification of the interface of **A** MTA, **B** 1% BG/MTA, **C** 2% BG/MTA, **D** 5% BG/MTA, and **E** 10% BG/MTA. The arrows indicate the precipitates in the dentinal tubules. 2% BG/MTA group showed denser precipitates in the dentinal tubules

Figure 2 shows the FE-SEM analysis results after the push-out bond strength measurements of the groups. In all groups, precipitates were observed in the dentinal tubules. Among all groups, the 2% BG/MTA group showed a denser precipitate infiltration (Fig. 2(C)).

### Compressive strength

**Fig. 3:**
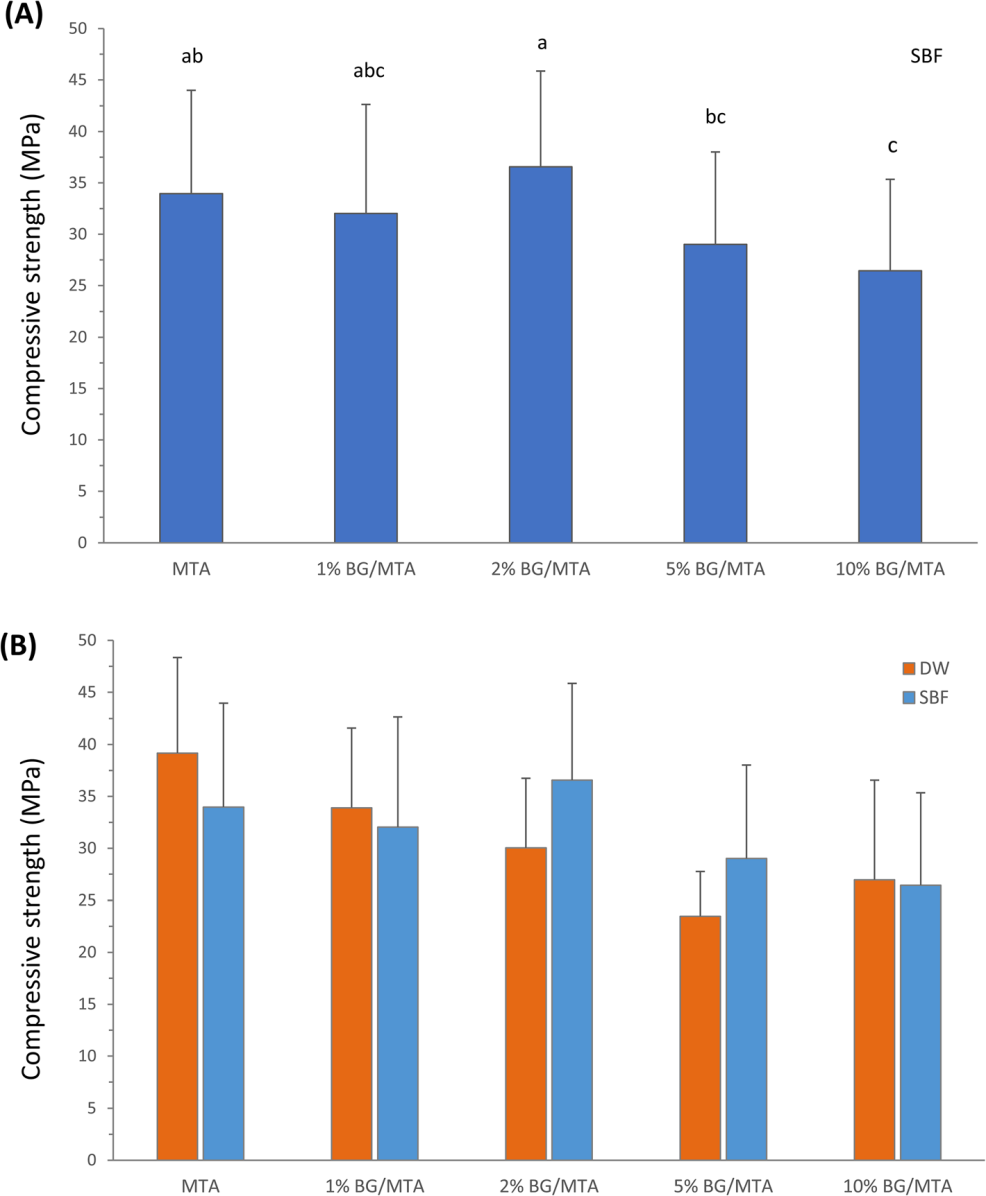
**A** Results of the compressive strength. Results of experimental groups in SBF storage solution for 7 days. Same superscript means no statistical significance (*p* > 0.05). **B** The results of experimental groups in comparison of DW and SBF as storage solutions

The compressive strength results are illustrated in Fig. 3. Among the specimens stored in SBF for 7 days, the 2% BG/MTA group showed the highest compressive strength (Fig. 3(A)). The control group and the 1 and 2% BG/MTA groups showed statistically higher intensities than that of the other BG/MTA groups (*p* < 0.05). Comparing the compressive strength results between DW and SBF that were storage solutions, the 2 and 5% BG/MTA groups showed a tendency to increase the compressive strength in SBF compared to DW (Fig. 3(B)). No significant interaction was observed between the materials and storage solution by two-way ANOVA (*p* > 0.05).

### Setting time

Table 2 showed the setting time results for the groups. Compared to the MTA group, the setting time of all experimental groups decreased. The reduction of setting time from the MTA group (428.3 min) were the 1, 2% BG/MTA groups (398.3 and 401.0 min) respectively.

**Table 2:** Setting time (min) of the groups

Groups	Setting time (min)
MTA	428.33 (11.93)
1% BG/MTA	398.33 (18.82)
2% BG/MTA	401.00 (26.89)
5% BG/MTA	406.02 (25.16)
10% BG/MTA	416.67 (42.52)

Values are written as means (standard deviation)

### Solubility

The solubility results are presented in Table 3. The solubility of the BG/MTA groups was slightly higher than that of the control group (*p* < 0.05).

**Table 3:** Solubility (%) of the groups

Groups	Solubility (%)
MTA	1.17 (0.19) ^c^
1% BG/MTA	1.70 (0.36) ^ab^
2% BG/MTA	1.10 (0.04) ^bc^
5% BG/MTA	1.25 (0.36) ^ab^
10% BG/MTA	1.34 (0.12) ^abc^

Values are written as means (standard deviation)

Different superscript letters indicate significant differences between the materials (*p* < 0.05)

### XRD analysis

**Fig. 4:**
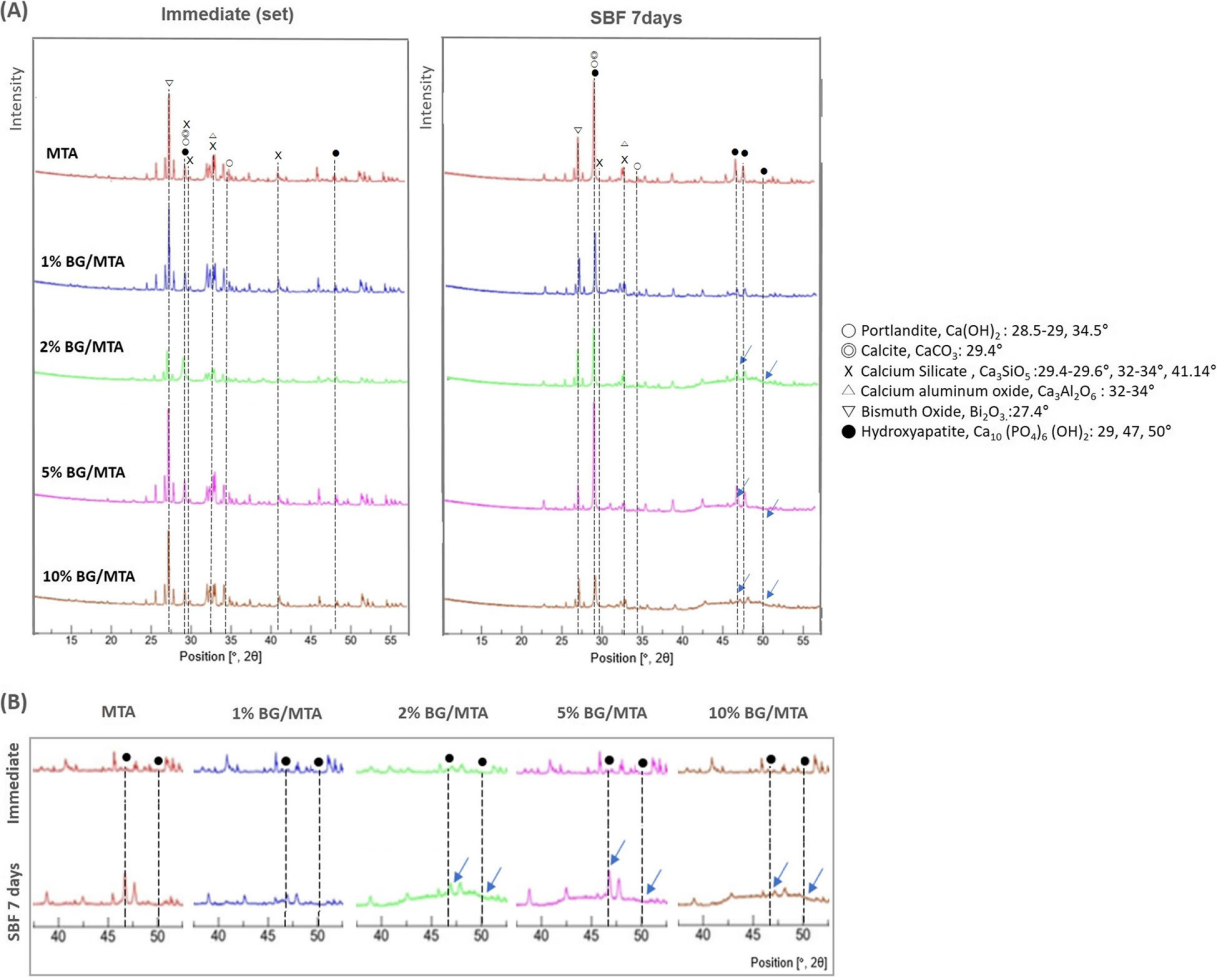
**A** XRD analysis graphs shows the difference between the analysis results of immediate after setting and 7-day storage in the SBF for each group. **B** In 2, 5, and 10% BG/MTA groups, the apatite peak was increased in each SBF 7-day storage specimen, compared to the control group immediately after setting (blue arrow)

The XRD analysis results are presented in Fig. 4. In the MTA group and 1% BG/MTA group, there was no difference between the analysis immediately after the setting and after the additional 7 days of SBF maturation in the graph. In the 2, 5, and 10% BG/MTA groups, the apatite peaks ranging from 40 to 50° in the graph were increased in each SBF 7-day maturation specimen, compared to the control group analysis immediately after the setting (Fig. 4(B)).

## Discussion

The biocompatibility and bioactivity of MTA have been demonstrated in a variety of endodontic treatment indications by laboratory and clinical studies. Though it has been widely used for 30 years, the disadvantage of decrease of physical properties, such as compressive strength, dimensional stability, and anti-washout property when in contact with periodontal tissue, blood, etc. is still one of the critical considerations [4, 7]. To overcome these characteristics, we examined BG, as an additive into MTA powder, which has been reported as a biomaterial with bone-like carbonated apatite-forming ability by the interaction between calcium ion and phosphate from tissue fluid. We evaluated the effect of BG on the physical properties of MTA, and demonstrated that the BG-containing MTA improved the physical properties in SBF condition or dentin bonding. We also investigated the optimal concentration of BG supplementation into MTA, which has an increasing effect or without hampering effect on the mechanical properties of MTA itself. Recently, several studies have been demonstrated the advantages of BG’s high biocompatibility and remineralization ability of dentin [19–23, 32]. Within our knowledge, a single previous study [11] had been reported that the addition of BG showed the shortening effect of the setting time of MTA Angelus and experimental Portland cement. They examined the effect of supplementation of various concentrations of BG and calcium metasilicate (wollastonite) ranged from 10 to 30 wt%. The study reported that BG addition over 10 wt% reduced the compressive strength of MTA, on the other hand, it has the shortening effect on the setting time without hampering the biological response. In our study, a significant important difference from the previous study [11] was found that supplementation of lower concentrations of BG which are 2 and/or 5 wt%, has the effect to increase both the push-out bond strength and the compressive strength in SBF storage condition.

The push-out bond strength test is an effective method for evaluating the ability to resist detachment from restored sites [33]; we attempted to find the evidence of the strength by analyzing the SEM results. All experimental groups showed increased push-out bond strength after 7 days of SBF storage, and the value of the 2% BG/MTA group was the highest (Fig. 2). FE-SEM (Fig. 3) following the push-out bond strength was not analyzed statistically; however, it consistently showed results that could predict uniform and dense precipitation in the 2% BG/MTA group. It is speculated that the immediate precipitation by the formation of an amorphous calcium-phosphate layer on the BG induces mineralization of the tag-like layer to the dentinal tubular interface during mineralization; the extrusion bond strength appears to increase [34]. BG as an additive improved the handling properties because of an appropriate amount of silica self-agglomeration [22] during the hydration process with DW, and thus, the deposition in the tubules increased. To investigate this, an additional energy dispersive X-ray spectroscopy (EDS) analysis is required to analyze the composition of the precipitate in the tubule. In our preliminary study, we evaluated several mechanical and biological properties of various concentration of BG into MTA ranged from 1 wt% to 20 wt%. We found that the supplementation of lower concentrations under 10 wt% of BG has the effect to increase both push-out bond strength to dentin and the compressive strength in SBF storage condition, especially in the 2 wt% BG/MTA showed the high push-out bond strength. In this study, we prepared the MTA or BG/MTA groups were prepared using the MTA and 63S BG at four different concentrations: 1, 2, 5, and 10 wt%. The L/P ratio of DW to the powder was 0.3 throughout the experiments, which was set according to the manufacturer’s recommendations. We supplemented the BG into MTA powder so that the L/P ratio of BG/MTA composite was 0.3, however, the pure MTA L/P ratio might be different. It is speculated that the BG might influence the MTA hydration and setting reaction, due to their ion exchange between BG and phosphate from SBF solution. In addition, the different shapes and size of particles between BG and MTA powder might lead to the different setting reactions of MTA in the SBF solution, followed by the different mechanical properties. Consequently, our results suggest that the critical optimal concentration of addition of BG, which could take biological advantages in the SBF condition and increasing the amount of hydroxyapatite by BG without hampering effect on the mechanical properties, might be considered ranged from 2 to 5 wt%.

Compressive strength is considered necessary in the evaluation of restorative MTA [35]. A comparison experiment was conducted to confirm the effectiveness of BG in SBF by dividing it into two storage solutions of DW and SBF. In the DW groups, the compressive strength decreased as the BG content increased, and the MTA group and 1 and 2% BG/MTA groups showed the highest values. The MTA and control groups only presented a significant decrease in SBF storage compared to the DW storage groups. Nekoofar et al. [9] reported that the compressive strength of MTA tends to decrease in SBF because of the decrease in the formation of acicular crystals. Therefore, it is speculated that this was the cause of the decrease in compressive strength in the SBF. In the SBF storage groups, the 2% BG/MTA group showed the highest value, and the 2 and 5% BG/MTA groups showed an increased compressive strength value compared to DW storage. The mineralization effect of BG compensated for the compressive strength of MTA, which was reduced in SBF. Skallevold et al. [18] reported that the glass composition of BG contributes to poor mechanical properties because of its fragility. Our results showed that the compressive strength of the 10% BG/MTA group, which did not increase further even under SBF conditions, may be responsible for the amount of fragile glass content rather than the increase in the amount of hydroxyapatite. The 27 mM HCO3- Tris SBF was used as the SBF storage solution in this study. The composition of 27 mM HCO3- Tris SBF is a component closer to plasma (27 mM HCO3- and 103 mM Cl-) than the original SBF (4.2 mM HCO3− and 148 mM Cl−) [30], and it had an enhanced ability to induce apatite-like calcium phosphate formation [23]. According to previous studies, there is a possibility of the autogenous precipitation of calcium phosphate when stored at 37 °C for several weeks [21, 36]; thus, SBF was exchanged once every 2 days in this study.

Setting time is a property for the stable maintenance of endodontic restorative materials. In this study, all BG/MTA groups showed a decrease in the setting time (Table 2). The initial hydration process of MTA occurs on the outer surface of the particles, and the rate of this process is affected by the particle size and the powder-to-liquid ratio that is determined from the setting time data. The particle sizes of MTA and BG used in this study ranged from to 1–30 μm [37] and < 20 μm [22], respectively. The effect of increased surface area and reactivity caused by the small size of BG [11, 38–40] could lead to a reduction in the setting time. The known setting time of the MTA is 4–6 h; however, the setting time of the experimental MTA was found to be 6–7 h (398–428 min), which is slightly longer. There are two types of specifications used for the method of measuring the setting time suggested in MTA-related studies [27]: ISO 6876, dental root canal sealing materials, and ISO 9917, and dentistry water-based cements. The ISO 6876 method is similar to the initial setting time because of the difference in the mold containing the specimen, and the ISO 9917 method is similar to the final setting time. We demonstrated along setting time by referring to the ISO 9917-1 method; however, it does not deviate significantly from the general range.

The solubility test is one of the most important requisites for endodontic restorative materials because it directly contacts the periodontal tissue fluids [41] and copes with the sealing of the wound. The solubility of all BG/MTA groups was slightly higher than that of the MTA group (Table 3); however, all groups showed values less than 3%, which is within the acceptable ISO 6876 range [31]. As the conditions for measuring the solubility of ISO were DW, calcium or phosphate ions could not be utilized in the surrounding solution [18, 42]. Therefore, it is assumed that a lower solubility value will be obtained if it is re-measured in a physiological solution environment where the action of BG can appear.

XRD was used to investigate the existence of hydroxyapatite formation in the experimental groups after 7 days of storage in SBF (Fig. 4); the 29° peak [34, 43] increased in the MTA, 1, 2, and 5% BG/MTA groups, and 47° and 50° peaks [34, 43] increased in the 2, 5, and 10% BG/MTA groups. Although there is a limitation in that XRD only analyzes surface components, it showed that the BG/MTA group can additionally form hydroxyapatite. However, the 2% BG/MTA group, which showed the highest compressive strength and push-out bond strength in SBF, did not exhibit the highest hydroxyapatite formation. From a clinical point of view, these results suggest that 2% BG/MTA improved the handling properties properly during the hydration process before setting; however, after setting, it contributed to the maintenance of physical properties by maintaining the appropriate amount of MTA along with an increase in the appropriate amount of hydroxyapatite by BG [18].

The possibility of reducing physical properties with BG at a high concentration of 10% or more can be inferred from previous studies dealing with GI or white Portland cement [11, 43]. However, to improve various physical properties at a specific concentration of 2% BG, further studies such as the component analysis of tubule precipitation are still required.

The biocompatibility of dental materials for perforation repair is an important characteristic. The MTA is a well-known biocompatible sealing material used for endodontic treatment [2]. The biocompatibility of BG has been proven to be an appropriate applicable material in the regeneration area [44, 45]. Although our investigation has not been performed, further study is necessary to evaluate the biocompatibility of BG-supplemented MTA, which includes viability, proliferation, and migration of dental pulp cells.

## Conclusion

Within the limitations of this study, it was verified that the BG-added MTA increased dentin push-out bond strength and compressive strength under SBF storage. There was no increase in setting time of MTA with BG, and the solubility was clinically acceptable within 3%. In SBF storage condition, increase hydroxyapatite leaks were shown in the BG-added MTA group. The addition of BG did not negatively affect the hydration and setting reactions of MTA, and as the production of hydroxyapatite was increased in SBF condition, MTA with BG could be considered to be utilized as a viable biocompatible restorative material for the clinical endodontics.

## Data Availability

The datasets during and/or analyzed during the current study available from the corresponding author or reasonable request.

## Abbreviations

BG: Bioactive glass
DW: Distilled water
EDTA: Ethylenediaminetetraacetic acid
MTA: Mineral trioxide aggregates
SBF: Simulating body fluid
XRD: X-ray diffraction
